# Use of barbed threads in facial rejuvenation

**Published:** 2008-10

**Authors:** Rakesh Kalra

**Affiliations:** Dept. of Cosmetic & Plastic Surgery, Ashirwad Hospital, Ashirwad Enclave, Dehradun-248 006, India

**Keywords:** barbed threads, barbs, cogs, thread lift, facial rejuvenation, APTOS, SILHOUETTE, CONTOUR

## Abstract

Use of barbed threads, available with uni- and bi-directional cogs or barbs, is a semi-invasive method of lifting sagging skin of the face. Areas treated with this method include the eyebrows, the cheeks, the jowls and the neck. Ease of use and a shorter down-time have made their use popular. Specific indications, operative procedures, risks and complications are described and some clinical results of the author shown.

## INTRODUCTION

As we age, our facial support structure weakens, and we lose facial fat. The affected areas generally include the cheeks, the eyebrows and other areas around the eyes, the jowls and the neck. The result is a longer, older-looking face. As the skin ages, the connective tissue in the skin becomes thinner. The elastic fibres in the skin undergo a type of “breakdown”. The face thus loses some of its elasticity. The lack of elasticity brings with it the departure of certain face-shaping supports. The face begins to “sag” and “wrinkle”. Younger people may experience cheek and brow ptosis (sagging, here caused by weakened muscles) as well.[1]

For these people especially, a thread lift may be a good alternative to the more invasive procedures necessary to correct problems in older people's faces. Thread lifts emerged recently because many people would like a facelift, but can't afford it or don't want the long recovery time of the standard facelift. It is, perhaps, better considered as a lesser, or preliminary procedure. Thread lifts cost less and require less downtime for many people. Some surgeons promote the thread lift as a “lunchtime lift” or “weekend facelift.” Usually it can be performed in about one hour.

### Place of barbed threads amongst the spectrum of procedures for non-surgical facial rejuvenation

A wide variety of procedures are being offered these days as non-surgical modalities for facial rejuvenation. These are:

- Botulinum toxin, which has a temporary effect lasting only three months.
- Fillers, which are limited to providing only limited volume and also last for about one to two years.
- Radio frequency which is limited to treating only very superficial wrinkles.
- Mesotherapy, which deposits so-called useful chemicals just under the skin, supposedly enhancing their utility in rejuvenating the skin colour and tone.
- Cosmetic camouflaging which is only a temporary measure to look good.

The thread lift provides an actual ‘lift’, and is a semi-invasive procedure. It has a long-lasting effect and in a milder form matches the effect of brow lift or a lower face and neck lift.

### The dynamics of thread lift

Barbs along the thread act as cogs to grasp, lift and suspend a relaxed facial area. The barbs open like an umbrella to form a support structure that lifts the sagging tissue. These barbs under the skin also tend to gather tissues to fill out and lift the cheeks and sagging skin.

**Figure 1:**
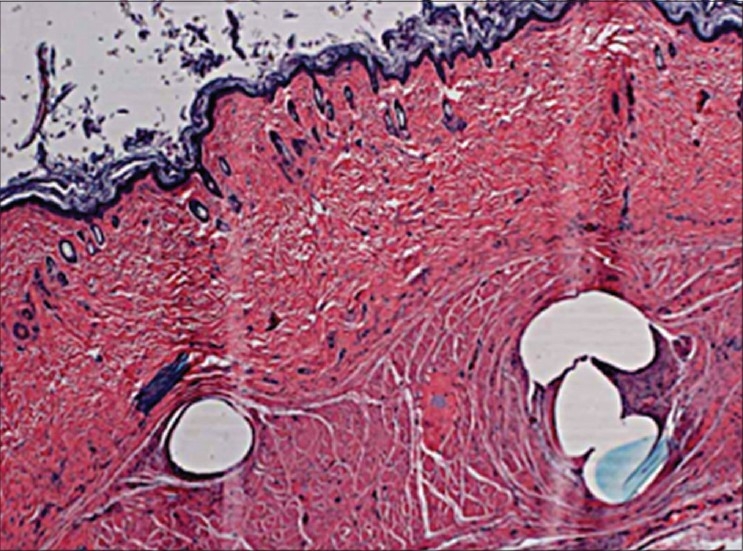
Histological slide showing collagen deposit around the thread and its knots (Source-Product Presentation “Silhouette Lift Sutures Suspension System” by Roberto Pizzamiglio M. D., Marbella-Spain and Franco Perego, M. D., Monza-Italy™)

Barbs on the thread grab hold of skin tissue. This creates tension in the thread, and the tension lifts the skin tissue. Collagen formation occurs around the threads and their cogs or barbs, producing an increasing effect[2,3] [Figure 1].

### Types of threads

**Figure 2:**
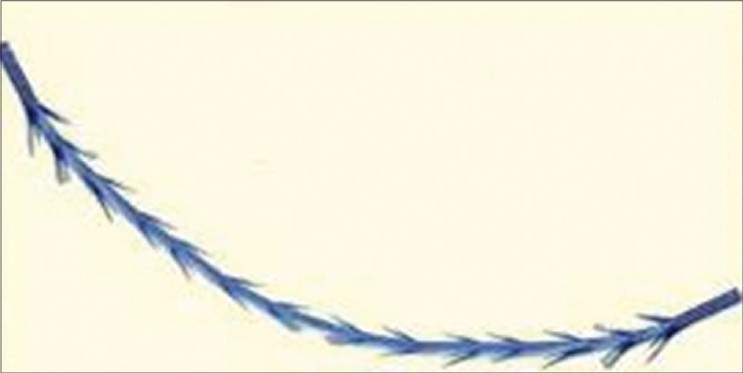
Bi- directional Barbs (Courtesy APTOS®)

**Figure 3:**
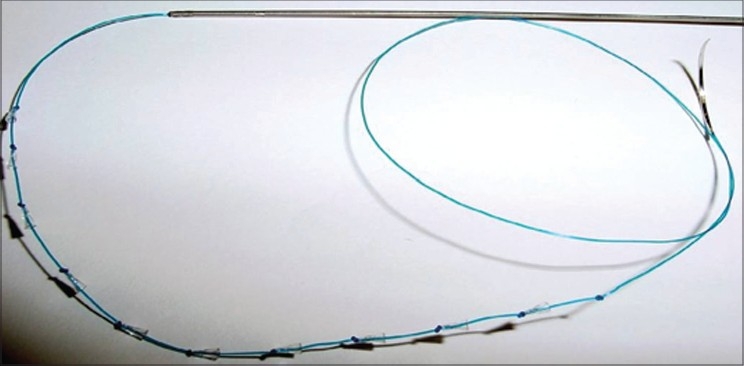
Uni-directional Cogs (Courtesy Silhouette®)

A large number of manufacturing companies, pioneers of thread lift and surgeons worldwide have their own patents as to not only the design of the threads, but also the minor variations of method of deploying them. However, essentially, there are two types of barbed threads which are available. These are:

- Bi-directional threads, with no anchoring points, inserted within a hollow needle and placed in such a manner that the thread cannot move either way because of the two-way direction of barbs fixing it nicely. Examples are the APTOS® threads
- Uni-directional barbed threads, which are anchored at a higher level fixation point. Examples are the Contour® and Silhouette® threads [Figures 2,3].

### Candidates for thread lift

#### Right candidates

Ideal candidates for thread lifts include people with minimal signs of ageing who need just a small lift. Usually, these are women between 35 and 45. The threads are indicated because these patients have begun to see more prominence of the jaw, a relaxed (or minimally sagging) mid-facial appearance or slight bags under the eyes or on the neck.

Older people may be advised to undergo a thread lift during the more aggressive facelift procedure to provide additional support for the soft tissue area that was elevated in the facelift.

Other thread lift candidates include those who have had some relapse from a previous procedure such as a facelift or neck lift.

Ideal patients are also those who understand and accept the possibility of the risks and complications outlined below, who understand the limitations of these threads, have realistic expectations and who are prepared to follow the post-operative regimen.

#### Poor candidates

Those patients who have excessively sagging skin at an advanced age will show a very limited improvement.

Those patients who are obese, or have very heavy, rugged skin too will show no improvement.

All other contraindications of implants, etc. also hold good here, like multiple skin allergic reactions or infections, dandruff, hair lice, immunologically compromising diseases like cancer/ HIV etc., systemic diseases like diabetes, tuberculosis, etc.

### Surgical procedure

#### Bi-directional threads

A classical example of their use is to lift the cheeks.

The anaesthesia chosen differs depending upon the patient, the surgeon and any associated procedures. When several other procedures are being done simultaneously, one can choose a general anaesthesia. When a lunch time thread lift alone has been planned, only a local anaesthetic can be infiltrated along the expected track. At most other times, if the patient is willing for a longer day care stay, twilight IV sedation with local anaesthetic is given. The last is the author's most preferred method.

A spinal needle is inserted under appropriate anaesthesia from the pre-tragal area, in a curved direction to a point below the cheek, and then up again towards a point stopping just short of the smile line. As this needle is advanced, it is actually progressed in a sinuous manner, moving the needle tip from side to side. Also, the depth of the needle is at all times maintained at the same sub dermal level, gliding just above the fat layer.

A similar needle is passed about one to one and a half centimetres above this line. Through the two needles, the bi-directional threads are inserted, making sure the thread and its barbs do not get ‘caught’ and do not damage.

Once the thread has been passed through, it is positioned such that the centre of the thread gets placed in the centre of the track. Once done, the needle is slowly withdrawn. The tissue can be ‘gathered’ over the thread, and once the desired effect has been reached, the long ends of the thread are then cut off [Figure 4].

**Figure 4a:**
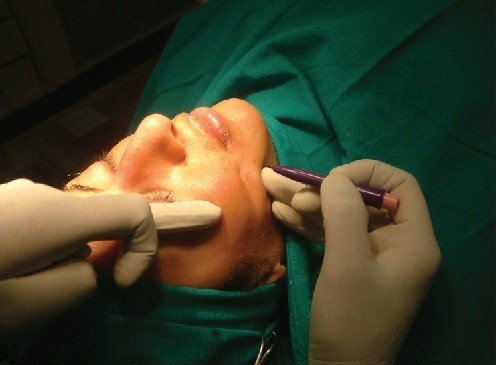
Defining the lowermost cheek point

**Figure 4b:**
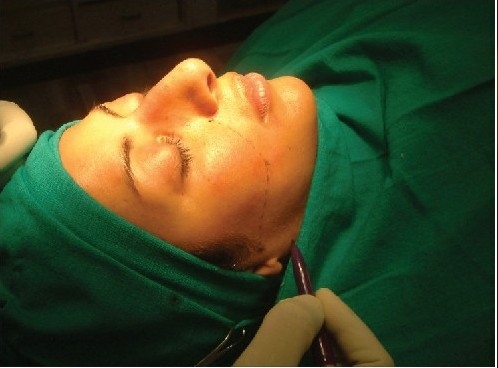
Marking the cheek curve

**Figure 4c:**
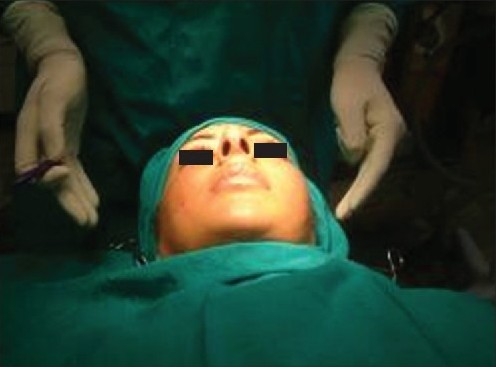
Ensuring bilaterally symmetrical markings

**Figure 4d:**
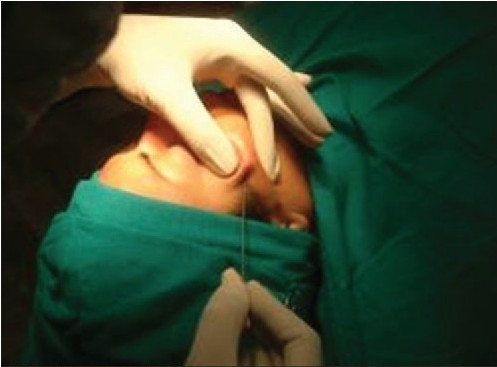
Inserting the spinal needle along the curve

**Figure 4e:**
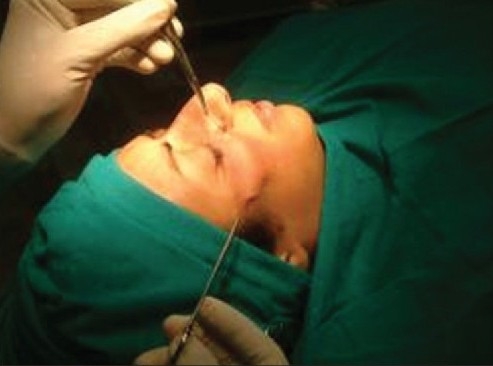
Thread firmly fixed, now does not budge on traction in either direction

**Figure 4f:**
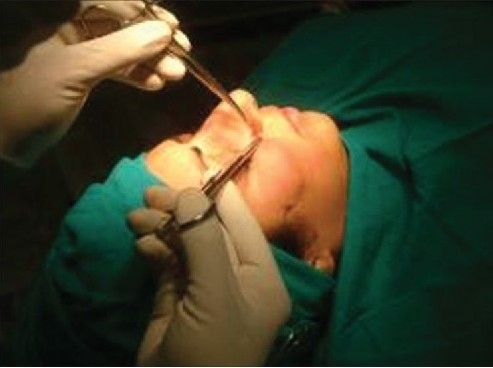
Thread snipped close to the skin

#### Uni-directional threads

Such threads are always placed in pairs. An equilateral triangle is marked in the temporal area, with its base parallel to the hair line, and the apex positioned more posterior. From the two corners of the line forming the base of the triangle, the uni-directional threads on their long needles are inserted, both parallel to each other, advancing at the same sub-dermal depth and in a sinuous manner, to points stopping just short of the smile lines [Figure 5].

**Figure 5a:**
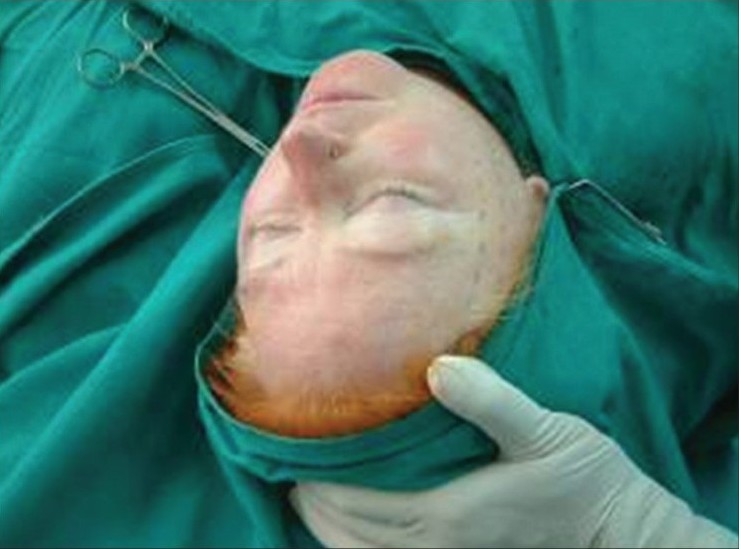
Parallel markings for a pair of threads for jowl lift

**Figure 5b:**
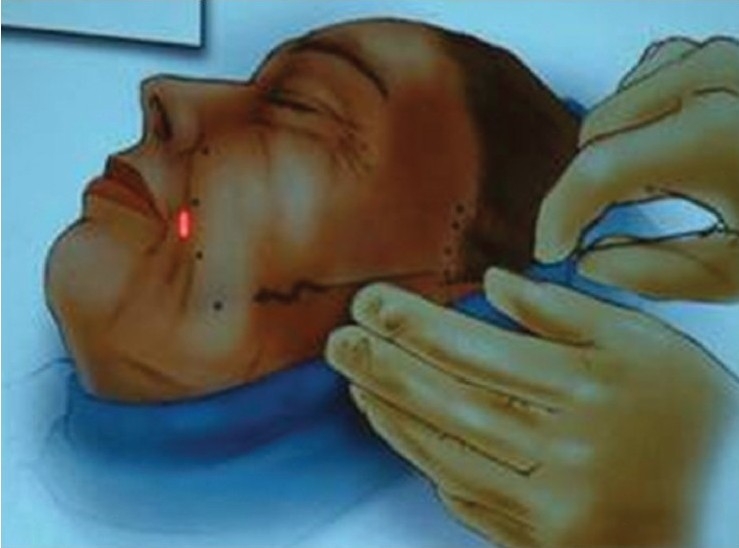
Zigzag manoeuvre to advance the barbed threads engaging maximum tissue (Picture courtesy Contour®)

The two threads in the temporal area are then brought out together from the third apical point, with the help of the curved cutting needle attached to the tail of the thread, this time taking a very deep sub temporal fascia bite. At this exit point, they are tied together, and the knot buried under the skin, so as to ensure that it is not palpable.

The threads at the smile line are left about an inch long, taped and the patient sent over to the recovery room. Once the patient has overcome all effects of any anaesthesia, maybe even on the second day, she is called back to help in the actual lift. While she holds up the mirror and watches herself, the surgeon holds the end of the thread firmly down and pushes the skin of the cheek back over it, gradually deploying more and more barbs. This is done to a desired effect, to which the patient consents. Once both sides are done, both the patient as well as the surgeon agree to the extent of the lift as well as symmetry, etc. Only then are the free ends actually snipped close to the skin. The area from the temporal area down to the cheeks is taped, in an upward supporting style, to prevent the patient from over animating and undoing the effect of the thread lift. These tapes are preferably left in for about a week [Figure 6].

**Figure 6a:**
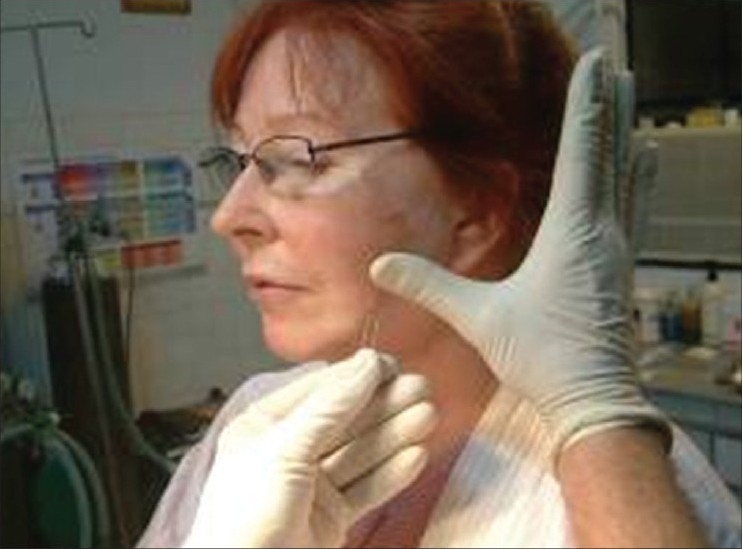
Deployment of the two threads by pulling the thread down and pushing the skin up, to the desired level

**Figure 6b:**
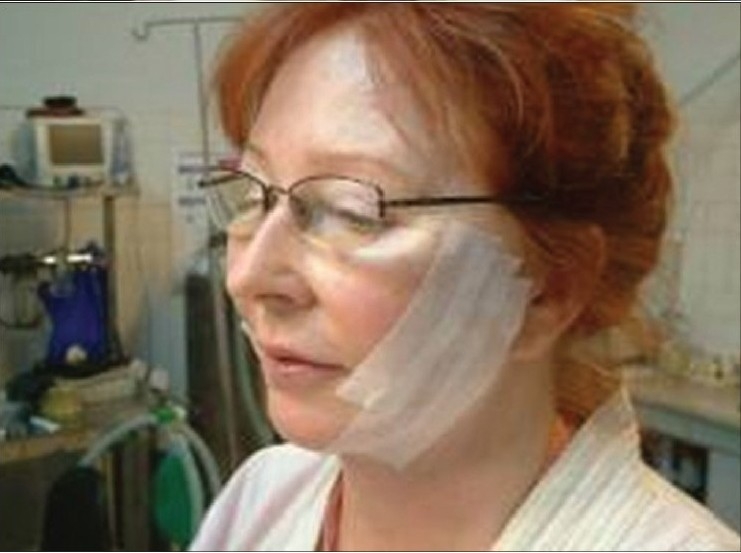
Taping at the end of the procedure to prevent undoing of the lift till some fibrosis occurs

### Recovery

Proper postoperative instructions must be given to reduce the risk of complications during recovery.

These instructions include:

Limited activities should be done for at least 24 hours.

Diet restrictions should be followed (soft foods) for seven days.

Pain can be managed with oral medications such as acetaminophen. Ibuprofen is avoided to limit bruising.

Though asepsis is strictly adhered to, it is preferable to give antibiotics for a period of five days as this is, after all, an insertion of a foreign body.

Recommend elevating the head on the first day to reduce swelling.

The day following the procedure, the patient can resume non-strenuous activities, and all normal activities can usually be resumed within seven days.

Social situations are to be avoided for up to one week — three weeks for weddings, reunions and other formal occasions.

### Postoperative care

Swelling and Bruising can be prevented by ice packs.

Movements are strictly restricted by taping the face, especially the area that has been operated. This is best advised for about a week.

Sleeping on the sides with the face against the pillow, can undo the effect of the procedure. Hence the patient is advised to sleep on the back for a week. Avoid excessive mouth opening and massage of any kind for three weeks.

### Risks and complications and their management[4]

The thread lift is a relatively new procedure, and its techniques are still being developed. Results have varied greatly among patients, but continue to improve.

A significant risk of the thread lift procedure is that one may not notice any improvement.

Asymmetry is a very bothersome consequence. One has to carry out the procedure with great precision, from noticing pre-procedure asymmetries, to perfectly balanced markings, to ensuring the symmetry with the patient's consent before cutting off the ends of the thread.

Some thread lift patients with thin skin have reported that the sutures became visible under the skin shortly after the procedure. Rippling can persist for long. The surgeons need to be aware of this problem, and have to be careful while inserting the thread so as to keep it at the optimum depth. This does require some experience, and hence there is a learning curve for this.

Some patients experience a lack of sensitivity or numbness in the treated area, which usually subsides within weeks of the procedure.

Infection inx the treatment area is an infrequent complication. If an infection develops, treat it with antibiotics. Rarely, an infection may require surgical drainage. Scar tissue formation is also possible. The knot of the thread in the scalp can cause formation of granulation tissue, bleed and be palpable. Sometimes, hair roots are pushed in deep, leading to hair sinuses and inclusion cysts.

Some surgeons have noted rare migration or even total extrusion of the sutures, causing an unbalanced facial appearance. With this, or if the thread breaks, a simple reinsertion solves the problem. If a very large bore cannula has been used to place bi-directional threads, then the track created is also very large-sized, and this can lead to an early extrusion of the thread [Figure 7].

**Figure 7a:**
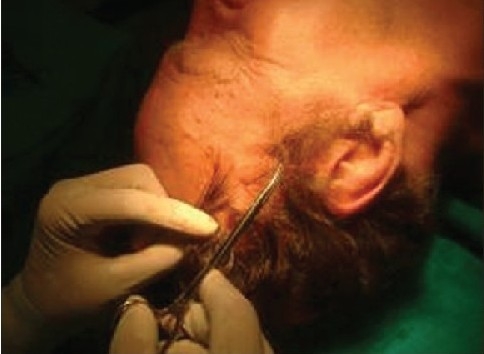
Hair sinus at site of entry

**Figure 7b:**
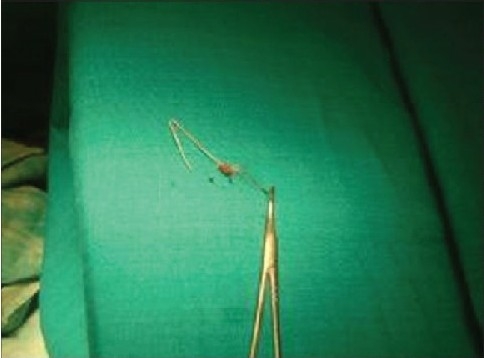
Hair root entangled within the knot of the thread

**Figure 7c:**
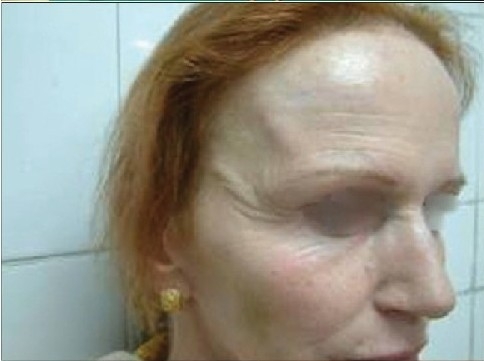
Unilateral puckering of skin requiring correction

**Figure 7d:**
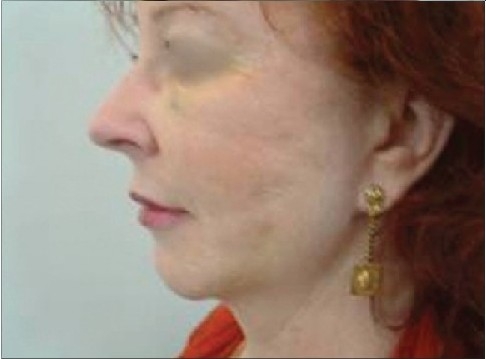
Rippling caused by too superficial placement of thread

**Figure 7e:**
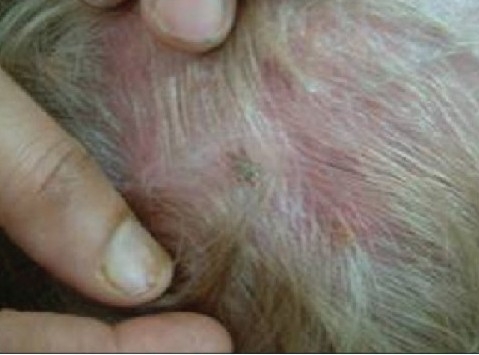
Crusting at the site of entry of thread

**Figure 7f:**
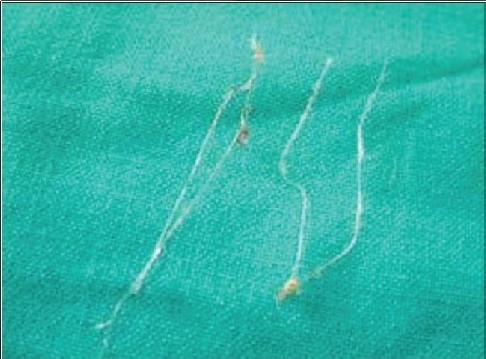
Infection causing extrusion of threads

**Figure 7g:**
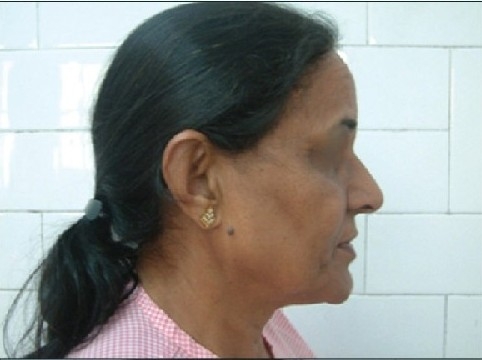
Before Thread Lift

**Figure 7h:**
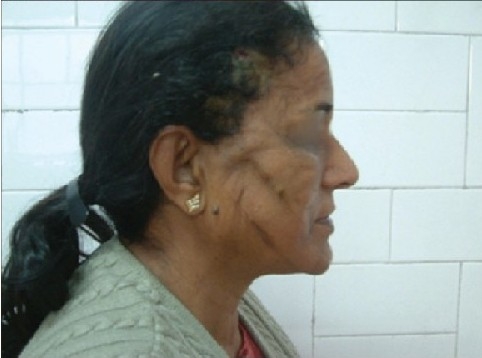
An extreme grade of rippling immediately following thread lift. The author had to undo the procdure

Some clinical cases of Thread Lift [Figure 8].

**Figure 8:**
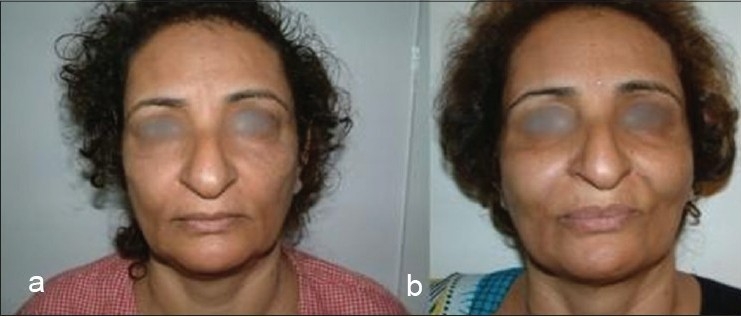
(a) Sagging cheeks (b) After Cheek Lift with two Bi-directional threads each side

**Figure 8:**
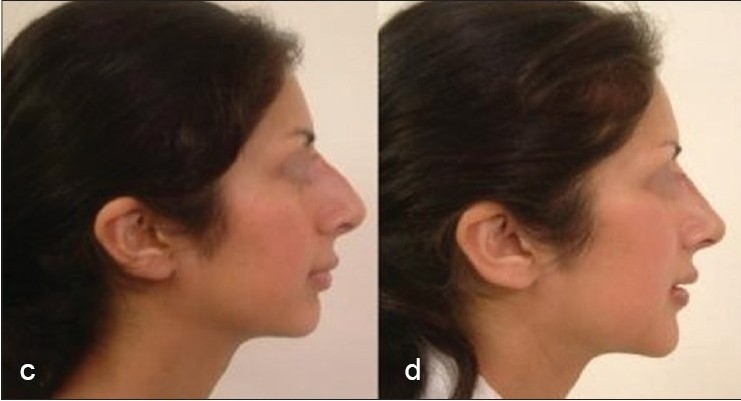
(c) Before profile improvement, (d) Besides rhinoplasty and chin augmentation, this girl was given a minimal, subtle cheek lift with threads only

**Figure 8:**
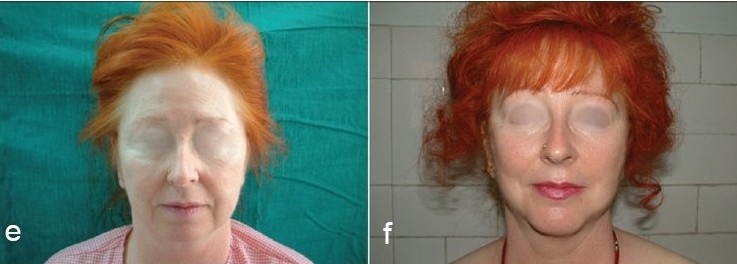
(e) Sagging cheeks and jowls (f) Cheek Lift and Jowls Lift by two pairs of Contour Threads™ each side (Other procedures done were lip augmentation/ chin augmentation and blepheroplasty)

**Figure 8:**
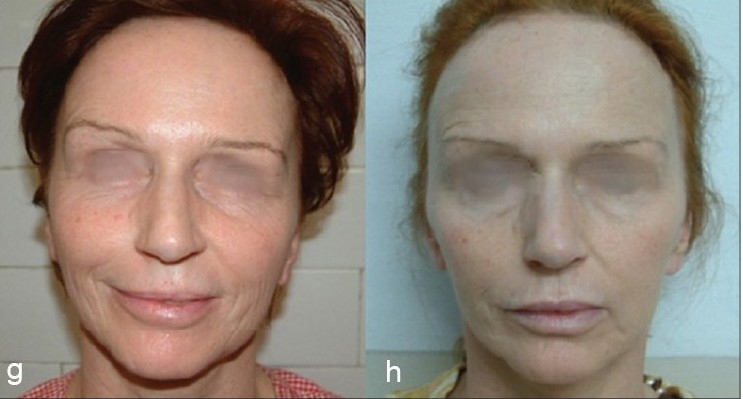
(g) Sagging Cheeks (h) Ogee curve created with Cheek Lift by Threads

**Figure 8:**
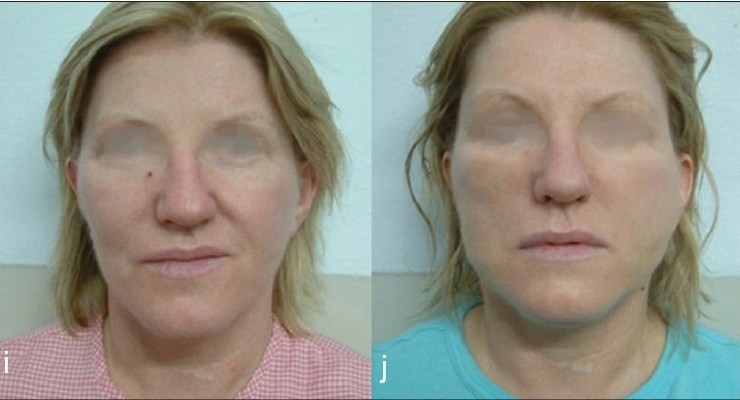
(i) Before Lift (j) After Thread lift, producing high cheek bone effect and reducing smile lines
